# Therapeutic effects of combined cell transplantation and locomotor training in rats with brain injury

**DOI:** 10.1038/s41536-019-0075-6

**Published:** 2019-06-05

**Authors:** Takafumi Shimogawa, Hideya Sakaguchi, Tetsuhiro Kikuchi, Ryosuke Tsuchimochi, Noritaka Sano, Sadaharu Torikoshi, Akira Ito, Tomoki Aoyama, Koji Iihara, Jun Takahashi

**Affiliations:** 1 Department of Clinical Application, Center for iPS Cell Research and Application, Kyoto University, Kyoto, Japan; 2 Department of Neurosurgery, Graduate School of Medical Sciences, Kyushu University, Fukuoka, Japan; 3 Department of Neurosurgery, Fukui Red Cross Hospital, Fukui, Japan; 4 Department of Physical Therapy, Human Health Sciences, Graduate School of Medicine, Kyoto University, Kyoto, Japan

**Keywords:** Regeneration and repair in the nervous system, Regenerative medicine

## Abstract

Cell-based therapies are attracting attention as alternative therapeutic options for brain damage. In this study, we investigated the therapeutic effect of a combined therapy of cell transplantation and locomotor training by evaluating the neuronal connectivity. We transplanted neural cells derived from the frontal cortex of E14.5 GFP-expressing mice into the frontal lobe of 3-week-old rats with brain injury, followed by treadmill training (TMT) for 14 days. In the TMT(−) group, graft-derived neurites were observed only in the striatum and internal capsule. In contrast, in the TMT(+) group, they were observed in the striatum, internal capsule, and the cerebral peduncle and spinal cord. The length of the longest neurite was significantly longer in the TMT(+) group than in the TMT(−) group. In the TMT(+) group, Synaptophysin^+^ vesicles on the neuronal fibers around the ipsilateral red nucleus were found, suggesting that neuronal fibers from the grafted cells formed synapses with the host neurons. A functional analysis of motor recovery using the foot fault test showed that, 1 week after the transplantation, the recovery was significantly better in the cell transplantation and TMT group than the cell transplantation only group. The percentage of cells expressing C-FOS was increased in the grafts in the TMT(+) group. In conclusion, TMT promoted neurite extensions from the grafted neural cells, and the combined therapy of cell transplantation and locomotor training might have the potential to promote the functional recovery of rats with brain injury compared to cell transplantation alone.

## Introduction

Damage to the cerebral motor cortex due to stroke or brain injury leads to motor dysfunction and a loss of coordination, often resulting in long-lasting nursing care.^1–5^ Current therapeutic options include surgery, drug administration, and rehabilitation as a physical therapy for the brain damage.

It has been shown that rehabilitation for brain damage reduces the impairment of motor functions and promotes compensatory functional recovery.^5^ Several reports have investigated the mechanisms of action of the rehabilitation, finding an elevation of neurotrophic factors and cytokines,^6,7^ the induction of neurogenesis,^8^ an enhancement of synaptic plasticity,^9,10^ the formation of compensatory neural circuits,^11–13^ and brain remapping.^11–13^ These rehabilitation-induced effects are considered to interact mutually and lead to an enhancement of brain repair, however, complete recovery by rehabilitation alone has low probability, and an alternative option is desired.

Currently, cell transplantation is expected to ameliorate the limited capacity of the damaged brain by regenerating some of the lost neural connectivity.^5,14–16^ Previous studies suggested that the transplantation of cells from the cerebral cortex can improve motor dysfunction.^15,17,18^ Neurite extensions, however, are not sufficient for substantial behavioral recovery, suggesting that an additional treatment is needed.^15,19^

A combined therapy with cell transplantation and rehabilitation is attracting attention as a therapeutic option after brain damage, since the effects of the rehabilitation on the host brain can affect the grafted cells.^5^ Previous studies suggested that the neuronal differentiation of grafted neural stem cells or mesenchymal stem cells is promoted by rehabilitation.^20–23^ These studies also demonstrated that the behavioral improvement of rodents with brain damage is enhanced by additional rehabilitation compared to cell transplantation alone. However, the effect of rehabilitation on neurite extensions from the grafted cells has not been examined, and the mechanism of the synergy effect remains unknown.

To address these issues, we investigated the neurite extensions of cells derived from the cerebral motor cortex of E14.5 mice grafted into the frontal lobe of rats with brain injury. We compared the effects of cell transplantation alone, locomotor training alone and the combination of both, and found that the combined therapy promotes neurite extensions from the grafted cells and functional recovery best.

## Results

### E14.5 mouse frontal cortex engrafted and extended neuronal fibers in lesioned rat brain

**Fig. 1:**
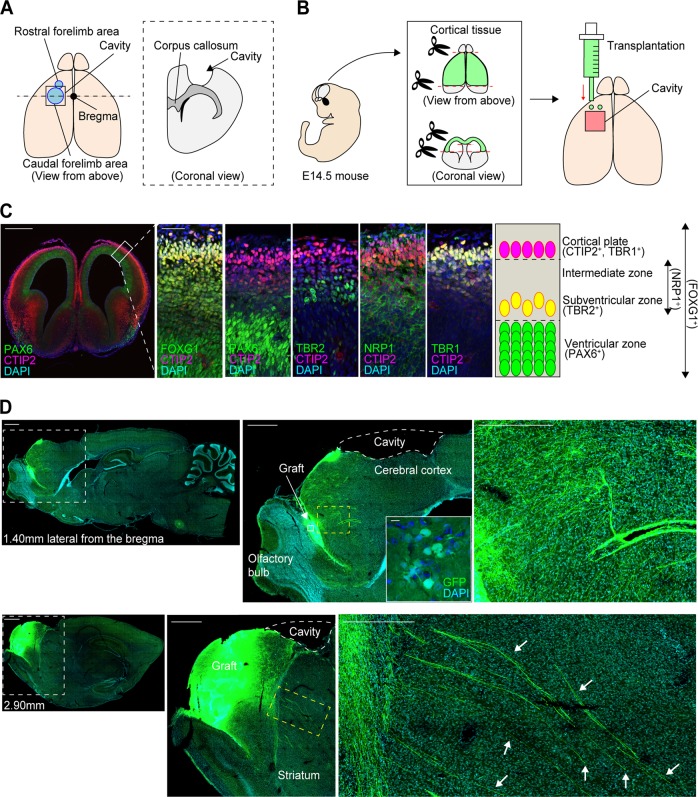
Schematic for the cortical lesions and cell transplantation, characteristics of the frontal cortex of E14.5 mice, and representative sagittal images after cell transplantation. **a** Schematic of the procedure for making cortical lesions. **b** Schematic of the cell transplantation procedure. E14.5 mouse cortical tissue is harvested and transplanted into the anterior part of the lesioned motor cortex. **c** Immunohistological evaluations of the anatomical cell distribution in E14.5 mouse cortex. FOXG1 is stained throughout the cortical tissues, PAX6 is stained mainly in the ventricular zone, TBR2 is stained outside of the ventricular zone, NRP1 is stained in a broad area of the tissues, including the intermediate zone, and CTIP2 and TBR1 are co-stained mainly in the cortical plate. Scale bars, 500 µm (left), 50 µm (right). Nuclear counter staining, 4,6-dimamidino-2-phenylindole (DAPI). **d** (Left images) Representative sagittal images of GFP staining at 1.40 mm and 2.90 mm lateral from the bregma at 2 weeks after the cell transplantation. Scale bars, 1000 µm. (Middle images) Magnified views of the white dotted frames in the left images (top), and magnified views of the white frames in the upper middle image (bottom). Scale bars, 100 µm. (Right images) Magnified views of the yellow dotted frames in the middle images. The fibers pass through the striatum (arrows). Scale bars, 50 µm. Nuclear counter staining, DAPI

As a brain injury model, the cerebral motor cortex (2 × 2 mm^2^) of a 2-week-old rat was aspirated 1 week before transplantation (Fig. 1a).^15^ Donor cells were prepared by dissecting the frontal cortex of E14.5 mice (Fig. 1b), because corticospinal motor neurons initiate axonal extensions at E13–14 in mice^24,25^ and the frontal cortex at this age contains neurons that contribute to the corticospinal tract (CST).^25,26^ We confirmed characteristics of the frontal cortex in E14.5 mice by an immunohistochemical analysis (Fig. 1c). A telencephalon marker, FOXG1, was expressed throughout the cortex, and a marker for cortical progenitor cells, PAX6, was expressed in the ventricular zone. An intermediate neural progenitor cell marker, TBR2, was expressed outside of the ventricular zone, and a marker for the initiation of axonal extension, NRP1, was expressed in a broad area, including the intermediate zone. Regarding cortical neuronal markers, a marker for layers 5 and 6, CTIP2, and a marker for layers 1 and 6, TBR1, were co-expressed in the cortical plate. These results indicated that the E14.5 frontal cortex contained several types of cells, including cortical projection neurons, intermediate migrating neurons and cortical progenitor cells. We transplanted the frontal cortex immediately after dissection and confirmed cell engraftment and neurite extensions from the grafted cells (Fig. 1d and Suppl. Fig. 1).

### Locomotor training promoted neurite extensions from grafted cells

**Fig. 2:**
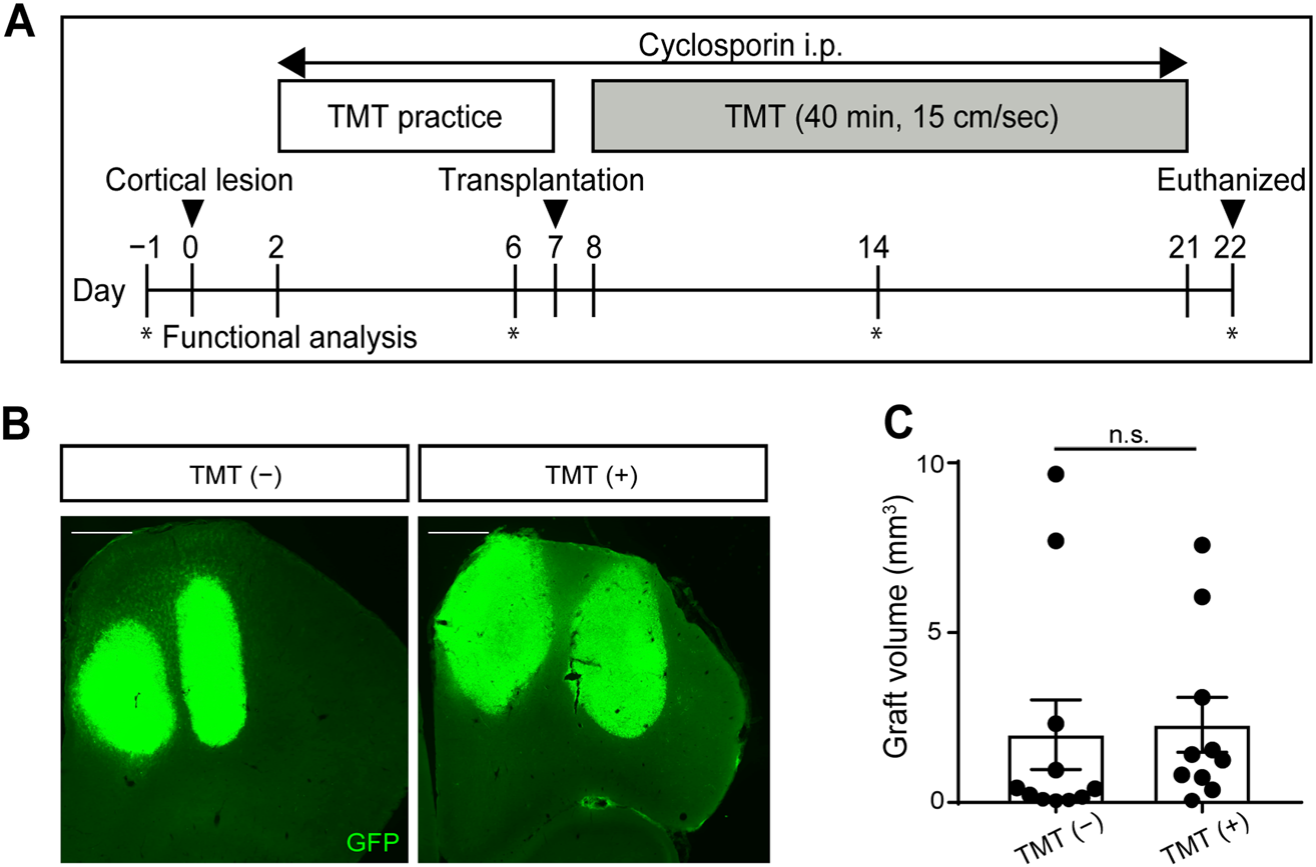
Schematic of the whole experiment and representative graft images. **a** Schematic of the whole experiment. **b** Representative coronal images of GFP staining at 2 weeks after cell transplantation without TMT (TMT (−) group) and with TMT (TMT (+) group). All specimens are shown in the section with maximum graft area. Scale bars, 500 µm. **c** Graft volume. There was no significant difference in graft volumes between groups. Mann–Whitney tests were performed; *n* = 11 in TMT (−) group and *n* = 10 in TMT (+) group. Data are presented as means ± SEM

To examine whether rehabilitation affects the outcome of the cell transplantation, we made the rats perform treadmill training (TMT) for 40 min (15 cm/s) daily until the day before euthanasia (Fig. 2a). After TMT practice for 5 days, four injections (1.0 µl of E14.5 brain tissue for each injection through two tracts) were performed into the frontal cortex adjacent to the injury area. Two weeks after the transplantation, we compared the graft volume between conditions with TMT (referred to as the TMT (+) group) or without TMT (referred to as the TMT (−) group). Cell survival was confirmed by immunostaining for GFP, and there was no significant difference of graft volume regardless of rehabilitation (Fig. 2b, c).

**Fig. 3:**
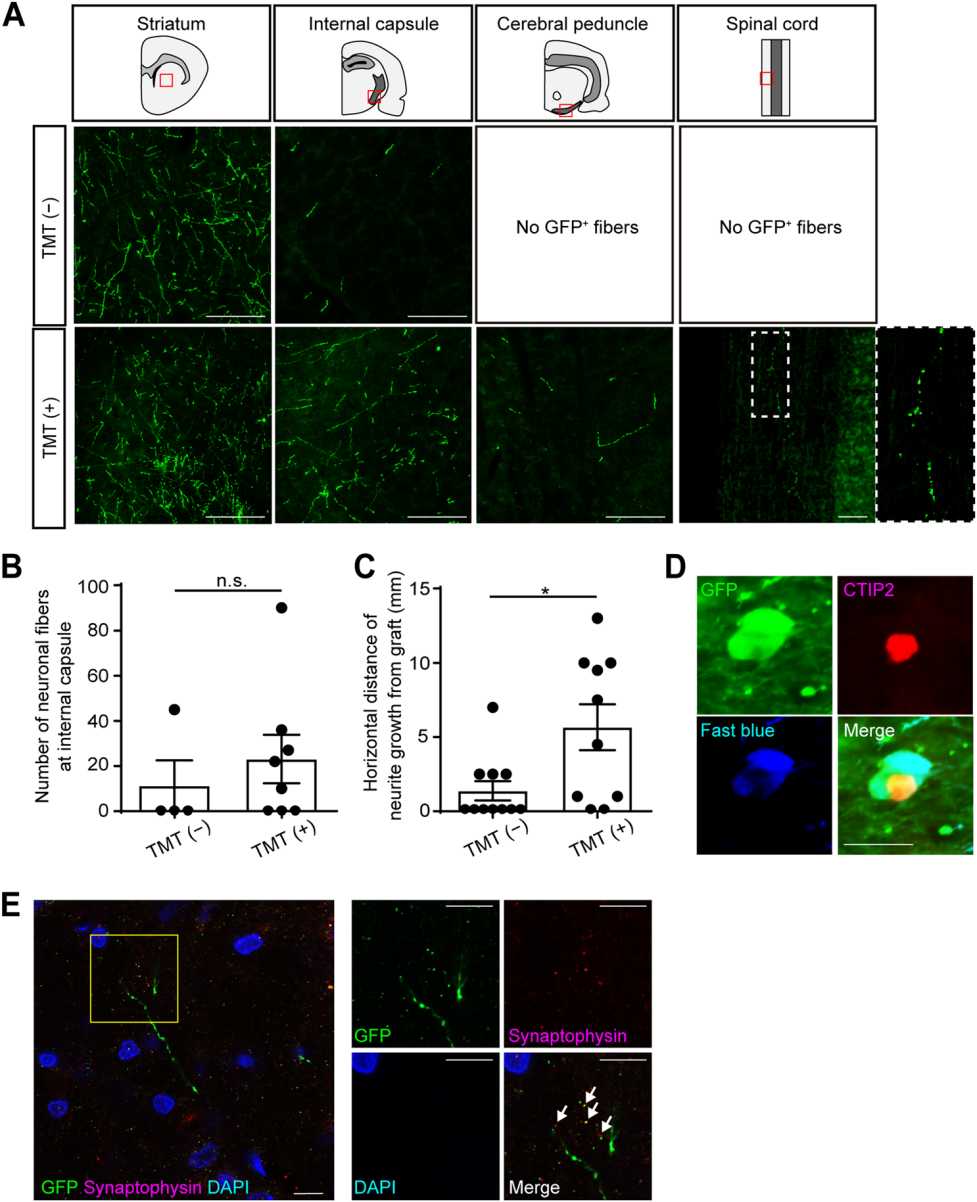
TMT promoted neurite extensions from grafted cells. **a** Representative images of GFP^+^ neurites at the ipsilateral striatum, ipsilateral internal capsule, ipsilateral cerebral peduncle, and spinal cord. Scale bars, 100 µm. In the TMT (−) group, extending GFP^+^ neuronal fibers were found in the ipsilateral striatum and ipsilateral internal capsule, but not in the ipsilateral cerebral peduncle or spinal cord. In the TMT (+) group, extending GFP^+^ neuronal fibers were found in the ipsilateral striatum, ipsilateral internal capsule, ipsilateral cerebral peduncle, and spinal cord. **b** Quantitative analysis of the number of GFP^+^ neurites at the ipsilateral internal capsule. The number of neurites was not significantly different between the TMT (−) group and the TMT (+) group. Mann–Whitney tests were performed; *n* = 4 in the TMT (−) group and *n* = 8 in the TMT (+) group. Data are presented as means ± SEM. **c** Quantitative analysis of the horizontal maximum distance of neuronal growth from the graft. The distance is significantly longer in the TMT (+) group than the TMT (−) group. Mann–Whitney tests were performed; *p* = 0.0219, *n* = 11 in the TMT (−) group and *n* = 10 in the TMT (+) group. Data are presented as means ± SEM. **d** Representative images of Fast blue^+^, GFP^+^ and CTIP2^+^ cells in the graft. In the TMT (+) group, double-labeled staining in the graft demonstrated that Fast blue^+^/GFP^+^ cells co-expressed CTIP2. Scale bar, 20 µm. **e** Representative images of GFP^+^ neurites and Synaptophysin^+^ vesicles on neuronal fibers around the ipsilateral red nucleus (arrows). Scale bars, 10 µm. Nuclear counter staining, 4,6-dimamidino-2-phenylindole (DAPI)

Next, we examined neurite extensions from the grafted cells (Fig. 1d and Suppl. Figs. 1 and 2). The neurites extended in all directions (Fig. 1d, upper column), and long fibers passed through the striatum (Fig. 1d, arrows), suggesting neurites extended along the CST. We could identify GFP^+^ neurites at the ipsilateral striatum, the internal capsule, the cerebral peduncle and the contralateral spinal cord by an immunohistochemical study (Fig. 3a). In the TMT (−) group, the neurites were observed only in the striatum and the internal capsule. On the other hand, in the TMT (+) group, the neurites were also found in the cerebral peduncle and the spinal cord (Fig. 3a). Furthermore, in the TMT (+) group, the neurites were observed in the corpus callosum, the ipsilateral cortex, and the ipsilateral red nucleus, which are other physiological targets of callosal and subcortical projection neurons (Suppl. Fig. 2a). To quantify neuronal outgrowth from the grafted cells, we counted the number of GFP^+^ neurites at the ipsilateral internal capsule. At the ipsilateral internal capsule, the number of neurites was not significantly different between the TMT (−) group and the TMT (+) group (Fig. 3b). Next, we measured the horizontal distance from the graft to the tip of the longest neurite in the sagittal plane (Suppl. Fig. 2b). The average distance was significantly longer in the TMT (+) group (5.67 ± 1.55 mm) than in the TMT (−) group (1.38 ± 0.65 mm) (Fig. 3c). These results indicated that TMT promoted neurite extensions from the grafted cells.

Neurite extensions from the grafts to the spinal cord were confirmed by the injection of a retrograde tracer, Fast blue, into the cervical spinal cord. Only in the TMT (+) group, could we find triple-labeled cells by GFP, CTIP2, and Fast blue in the graft (Fig. 3d), indicating that the grafted subcortical projection neurons extended neurites to the spinal cord.

We also found Synaptophysin^+^ vesicles on the neuronal fibers around the ipsilateral red nucleus (Fig. 3e), suggesting that neuronal fibers from the grafted cells formed synapses with the host neurons.

### Effect of combined therapy toward promotion of motor functional recovery

**Fig. 4:**
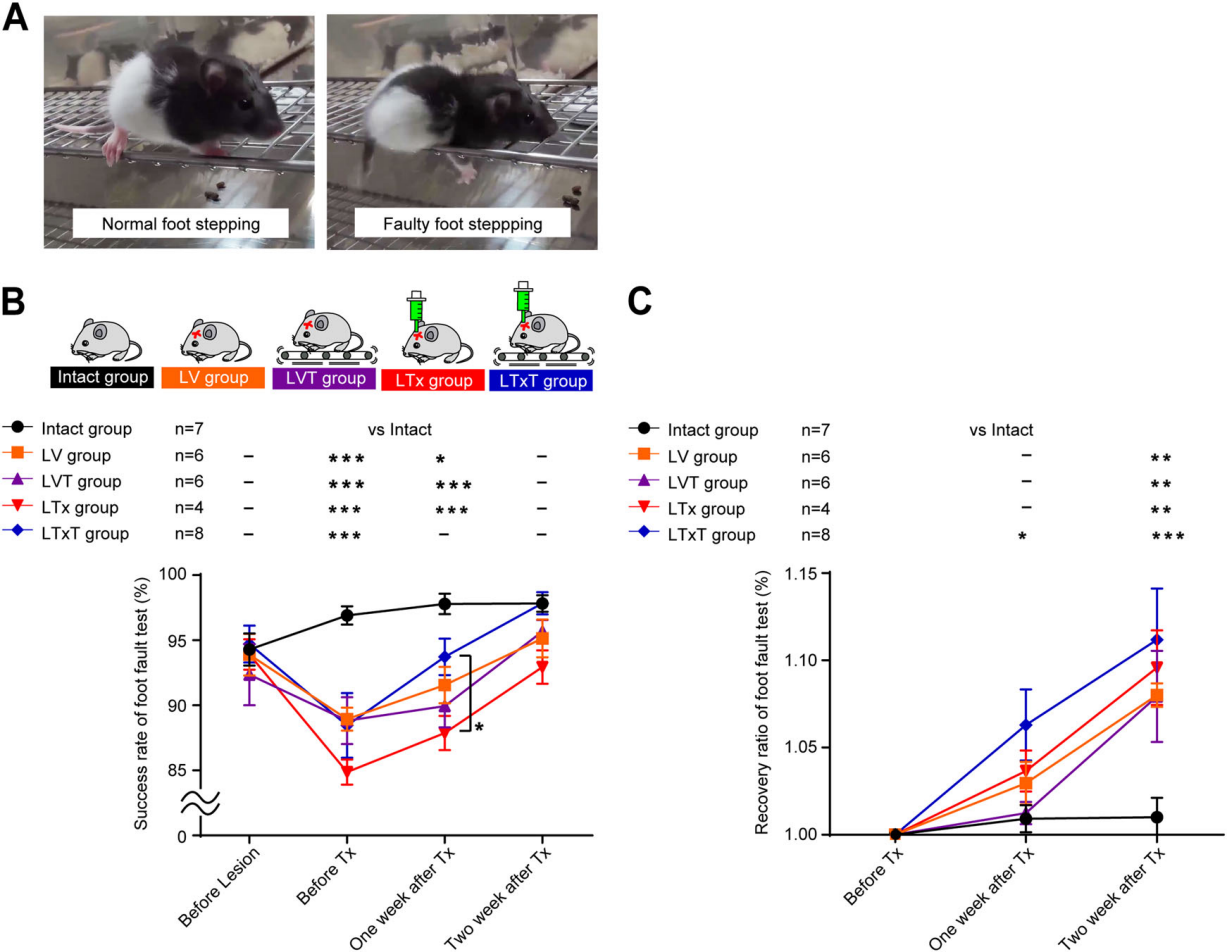
Effect of combined therapy toward promotion of motor functional recovery. **a** Representative captures of normal foot stepping and faulty foot stepping states from the video recordings. **b** The average success rates of the foot fault test for the intact group (*n* = 7), lesion and vehicle injection group (LV group; *n* = 6), lesion and vehicle injection with TMT group (LVT group; *n* = 6), lesion and cell transplantation group (LTx group; *n* = 4) and lesion and cell transplantation with TMT group (LTxT group; *n* = 8) are shown. Before making the lesion, the rates were similar for all five groups. One week after the transplantation, the success rate was significantly higher in the LTxT group than the LTx group. Two-way ANOVA with Tukey’s multiple comparisons test; *p *<* 0.05, **p *<* 0.01, ***p *<* 0.001. Data are presented as means ± SEM. **c** Average recovery ratios in the foot fault test for the intact group, lesion and LV group, LVT group, LTx group, and LTxT group are shown. Recovery ratios are defined as the success rate of the foot fault test compared to the baseline score just before the cell transplantation in each group. The recovery ratio of the motor function was higher in the LTxT group compared to the intact group at 1 week after the transplantation, however, it was not different compared to the LV group at 1 week or 2 weeks after the transplantation. Two-way ANOVA with Tukey’s multiple comparisons test;, *p *<* 0.05, **p *<* 0.01, ***p *<* 0.001. Data are presented as means ± SEM

To investigate whether cell transplantation and TMT improve the motor function of rats, we performed a foot fault test (Fig. 4a) and evaluated the success rate of foot stepping in five groups: intact group (*n* = 7), lesion and vehicle injection group (LV; *n* = 6), lesion and vehicle injection with TMT group (LVT; *n* = 6), lesion and transplantation group (LTx; *n* = 11) and lesion and transplantation with TMT group (LTxT; *n* = 10). Brain injury correlated with poor performance in the foot fault test, but it gradually improved over 2 weeks (Fig. 4b and Suppl. Fig. 3). One week after the transplantation, the success rate was significantly higher in the LTxT group than the LTx group (Fig. 4b). When compared to the LV group, however, there was no significant difference at 1 and 2 weeks after the transplantation (Fig. 4b). These results indicated that the combined therapy might have the potential to promote functional recovery compared to cell transplantation alone, at least during the very early stage of the therapy.

### Neuronal activity in grafted cells was increased by locomotor training

**Fig. 5:**
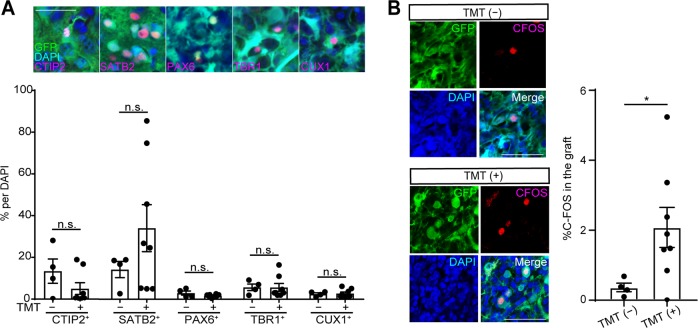
Activation of grafted cells by locomotor training. **a** Representative images of cells expressing CTIP2, SATB2, PAX6, TBR1, and CUX1 in the GFP^+^ graft in the TMT (+) group. Scale bar, 50 µm. Nuclear counter staining, 4,6-dimamidino-2-phenylindole (DAPI). Quantitative analysis of cells expressing CTIP2, SATB2, PAX6, TBR1, and CUX1 in the graft showed there were no differences in the percentages of CTIP2^+^ cells, SATB2^+^ cells, PAX6^+^ cells, TBR1^+^ cells, and CUX1^+^ cells per DAPI^+^ cells between the TMT (−) group and TMT (+) group. Mann–Whitney tests were performed; *n* = 4 in the TMT (−) group and *n* = 8 in the TMT (+) group. Data are presented as means ± SEM. **b** Representative images of double-labeled stained cells of C-FOS^+^ and GFP^+^ cells in the graft. Scale bars, 50 µm. Nuclear counter staining, DAPI. Quantitative analyses of the cells expressing C-FOS in the graft showed the percentage of C-FOS^+^ cells was significantly higher in the TMT (+) group (2.08 ± 0.57%) than in the TMT (−) group (0.36 ± 0.12%). Mann–Whitney tests were performed; *n* = 4 in the TMT (−) group and *n* = 8 in the TMT (+) group. Data are presented as means ± SEM

The results above indicate that the combined therapy of cell transplantation and locomotor training promoted the functional recovery of motor dysfunction caused by brain injury compared to cell transplantation alone. To investigate the effect of TMT on the grafted cells, we examined the components of the grafts in the TMT (−) and TMT (+) groups by an immunohistochemical staining 2 weeks after the transplantation. The most frequent type of cells observed were SATB2^+^ callosal projection neurons, which accounted for 14.25 ± 3.88% and 34.09 ± 11.26% of the grafts in the TMT (−) and TMT (+) groups, respectively. CTIP2^+^ subcortical projection neurons, which contribute to the CST, accounted for 13.4 ± 5.82% and 5.03 ± 2.82% of the grafts, respectively (no significant difference). Similarly, there were no significant differences in SATB2^+^, PAX6^+^, TBR1^+^ and CUX1^+^ (upper layer neurons) cells (Fig. 5a). Therefore, it is not likely that TMT increased the number of subcortical projection neurons in the grafts.

We then investigated whether the neuronal activity of the grafted cells changed after TMT. To confirm this possibility, we examined the expression of C-FOS, which plays a role in sprouting responses associated with neuronal activity.^27^ Double-labeled immunofluorescence staining of the grafts revealed that the percentage of C-FOS^+^ cells (%C-FOS) was significantly higher in the TMT (+) group (2.08 ± 0.57%) than in the TMT (−) group (0.36 ± 0.12%) (Fig. 5b), indicating that the neuronal activity in the grafted cells was increased by locomotor training.

## Discussion

We demonstrated that the combined therapy of cell transplantation and locomotor training promoted neurite extensions from grafted cells and might have the potential to promote the functional recovery of rats with brain injury compared to cell transplantation alone. The success of cell transplantation depends on the ability of the grafted neurons to survive, differentiate, integrate into the host’s neuronal networks, and reconstruct the damaged pathways.^15^ Therefore, we hypothesize that our combined therapy will contribute to a cell-based therapy for motor dysfunction caused by stroke or brain injury.

In the present study, we revealed two important findings. First, TMT promoted neurite extensions from the grafted cells (Fig. 3a, c). We found neurite extensions were limited to the internal capsule in the TMT (−) group, but expanded to the spinal cord in the TMT (+) group. Second, the combined therapy might have the potential to promote functional recovery from motor dysfunction compared to cell transplantation alone (Fig. 4b, c). Previous studies reported that rehabilitation causes an increase of neurotrophic factors and cytokines,^6,7^ an enhancement of synaptic plasticity,^9,10^ an increase in blood flow and an enhancement of angiogenesis^22,23^ in the damaged brain. The increase of neurotrophic factors and the enhancement of synaptic plasticity can promote cellular migration after cell transplantation,^20^ and an enhancement of angiogenesis in the host brain can promote cellular differentiation.^22,23^ An increased blood supply to the graft is essential for better graft survival and neurite extension after cell transplantation.^15,16^ Therefore, we speculate that neurotrophic factors, synaptic plasticity and blood supply might be enhanced by TMT to improve neurite extension and functional recovery. We also showed that TMT increased %C-FOS in the grafts (Fig. 5b). If neurotrophic factors or cytokines are increased in the brain after TMT, they upregulate immediate early genes, including C-FOS.^28^ This signaling pathway is essential for establishing neural connectivity and evoking action potentials in neuronal cells.^29^ Indeed, in our experiments, in the TMT (+) group, neuronal fiber extensions were longer and synapse formation was observed. Therefore, we concluded that the C-FOS expression in the grafts might reflect neuronal activity in response to a sustained expression of neurotrophic factors or cytokines.

The contribution of neuronal circuits outside the CST should be considered as another possibility for the effect of the combined therapy. Previous studies reported that cortico-striatal projections, cortico-thalamic projections, cortico-rubral projections, and contralateral axonal rewiring from the intact cortex were all increased after brain damage,^13,30^ and these alternative pathways could be enhanced by rehabilitation to contribute to functional recovery.^13^ In our study, neurites from the grafts were observed in the corpus callosum, ipsilateral cerebral cortex and ipsilateral red nucleus, but not in the contralateral cortex of the TMT (+) group (Suppl. Fig. 2a). Moreover, Synaptophysin^+^ vesicles were observed on the neuronal fibers around the ipsilateral red nucleus of the TMT (+) group by immunohistochemical staining 2 weeks after the transplantation (Fig. 3e), indicating that the neuronal fibers from the grafted cells formed synapses with the host neurons at the ipsilateral red nucleus. On the other hand, neurites were not found in the red nucleus of the TMT (−) group. From these data, in addition to reconstruction of the CST, we speculate that the ipsilateral cortico-rubral pathway might contribute to the functional recovery in the TMT (+) group. To investigate alternative pathways, retro/antero tracing or three-dimensional (3D) imaging of the neural circuits with the combined therapy would be useful.

Although we demonstrated advantages in our combined therapy, there still are limitations in our study. The most critical point is that we evaluated motor function only by a foot fault test and could not obtain more detailed functional evaluation. In our study, we used 3-week-old rats as the host because graft survival was poorer in older rats (Suppl. Fig. 4), but these infant rats are not suitable for other complex analyses, which often need long-time practice. It is reported that an intrinsic program for neuronal outgrowth affected by growth factor, cell adhesion, axonal guidance and cytoskeletal-modifying molecules differs with the age of the animal and the duration after the brain damage.^31^ Age-related differences in cell adhesion and axonal guidance molecules might play a crucial role in the survival and neurite extension of the grafted cells. Therefore, it is possible that the effect of TMT might be different in older rat models. In addition, to clarify the effectiveness of the combined therapy for motor functional recovery, we need to modify the dose of TMT and examine the long-term behavioral analysis.

Considering the rapid progress of stem cell technology, it is expected a cell-based therapy will eventually be used to treat patients with brain damage. As shown here, the function of grafted cells can be enhanced by locomotor training. We hypothesize that a combined therapy with cell transplantation and locomotor training represents a promising therapeutic strategy for the treatment of brain damage, including stroke and brain injury.

## Methods

### Animals

All procedures involving animals were approved by the guidelines for animal experiments of Kyoto University and the guide for the care and use of laboratory animals of the Institute of Laboratory Animal Resources (ILAR; Washington, DC, USA). All attempts were made to minimize the suffering and the number of animals used in this study. Male Crlj:LE rats were group-housed under a 12 h light and dark cycle, with food and water available. A total of 76 rats were used in this study as follows: 57 rats were lesioned, transplanted and used for the neuroanatomical and behavioral study (37 two-week-old rats, 10 seven-week-old rats, 10 twelve-week-old rats), 12 two-week-old rats were lesioned and used for the behavioral study, and 7 two-week-old rats were used as control in the behavioral study. A total of 80 C57BL/6-Tg[CAG-EGFP] mouse fetuses of either sex^32^ were used to acquire graft tissues. All rats were purchased from Charles River Laboratories Japan, Inc. (Yokohama, Japan), and all mice were from Japan SLC (Shizuoka, Japan).

### Animal model with lesion in motor cortex area

Rats were anesthetized with isoflurane (1.5%) in a mixture of O_2_ and N_2_O (50%: 50%) and clamped in a stereotactic apparatus to keep their head at the horizontal position (Narishige, Tokyo, Japan). After checking for the absence of the pain reflex by pinching the cranial skin, a small midline incision in the skin and a small window of the skull over the motor cortex was made using a drill (Minitor Co., Tokyo, Japan). The motor cortex was aspirated from −1.0 to 1.0 mm rostral to the bregma and from 0.5 to 2.5 mm lateral to the midline in 2-week-old rats with the corpus callosum left intact, and −1.5 to 1.5 mm rostral to the bregma and from 0.5 to 3.5 mm lateral to the midline in 7- and 12-week-old rats (Fig. 1a). Body temperature was maintained in the normothermic range (37–38 °C) with a feedback-controlled heating pad and incubator (Biomachinery Co., Chiba, Japan).

### Cortical tissue harvesting and transplantation

The cortical tissues of E14.5 mice were harvested and transferred to HBSS (Gibco) and kept at 4 °C until the transplantation. Then, the tissues were transplanted into the anterior part of the lesioned motor cortex of 3-, 8-, and 13-week-old rats 1 week after the lesion.^15,25,26^ Rats were anesthetized and clamped with the same methods, and a small craniectomy in the anterior part of the lesion was made using the drill. The mouse tissues were sucked up with a sterile 22 gauge needle and transplanted into four sites (1.0 µl/site) targeting the frontal cortex in the anterior part of the lesion (from the bregma: anterior 1.5 mm, lateral 1.0 mm and 2.0 mm, vertical 0.5 mm and 1.0 mm in 3-week-old rats; anterior 2.0 mm, lateral 1.0 mm and 3.0 mm, vertical 1.0 and 1.5 mm in 8- and 13-week-old rats). The injection was held for 1 min or longer (Fig. 1b).^33^ The rats received intraperitoneal injections of the immunosuppressant Cyclosporin A (10 mg/kg) (Wako) every day starting 5 days before the transplantation until the day before euthanization. As older rats showed poorer graft survival, we decided to use 3-week-old rat as the host for the remaining experiments (Suppl. Fig. 4).

### Behavioral analysis

We examined the motor functional recovery after treatment using the foot fault test. The apparatus consisted of a grid floor (1.5 cm × 1.5 cm per grid). Each rat was placed at one end of the grid and monitored by video recording for 2 min as they traversed the grid. The number of affected forelimb and hindlimb placement errors was scored, and the percentage of foot faults out of total steps was calculated.^34,35^ The test was performed the day before the lesion, before the cell transplantation, and 1 week and 2 weeks after the transplantation. Baseline scores of intact 2-week-old rats (*n* = 7) were recorded. The other rats were randomly divided into the following four experimental groups: lesion and vehicle injection group (LV; *n* = 6), lesion and vehicle injection with TMT group (LVT; *n* = 6), lesion and transplantation group (LTx; *n* = 11) and lesion and transplantation with TMT group (LTxT; *n* = 10). Seven rats out of the LTx group and two rats out of the LTxT group were excluded from the analysis because the transplanted tissues did not engraft sufficiently according to immunohistochemical analyses. An observer blinded of the therapeutic interventions performed the behavioral analysis by video recordings.

### Treadmill training

TMT was used as the rehabilitation therapy (Harvard Apparatus, MA, US). We set the time period of TMT to 2 weeks after the transplantation, because cortical neuroblasts transplanted into damaged adult motor cortex developed appropriate projections to cortical and subcortical targets 2 weeks after grafting.^36^ Next, we set the speed of the TMT to 15 cm/s, because 3-week-old rats could not run above this velocity over a long-time. Finally, we determined the effective TMT time as follows. We divided 16 rats into four equally sized groups as lesion only group, lesion + 20 min TMT (TMT 20 min group), lesion + 40 min TMT (TMT 40 min group), and lesion + 60 min TMT (TMT 60 min group) (*n* = 4, respectively). Two days after the lesion, rats began treadmill running 10 min/day at a speed 10 cm/s and slope of 0 degrees. For successive days, the running speed was increased 1 cm/s every day until 1 week after the lesion. Then, TMT 20 min, TMT 40 min, and TMT 60 min groups started TMT at 15 cm/s for 1 week. The foot fault test was performed at the day before the lesion, and 1 week and 2 weeks after the lesion. At 2 weeks after the lesion, the success rate was significantly higher in only the TMT 40 min group compared with the lesion only group (Suppl. Fig. 5). Therefore, the TMT group in our experiments is represented by the TMT 40 min group. In our experiment (Fig. 2a), 2 days after the lesion, rats began treadmill running 10 min for 5 days at a speed 10 cm/s and slope of 0 degrees. The running speed was increased 1 cm/s every day until the transplantation. One day after the transplantation, the rats started TMT at 15 cm/s for 40 min.

### Immunostaining

Fourteen days after the transplantation, rats were transcardially perfusion-fixed with 4% paraformaldehyde (PFA) (Wako), and brains and spinal cords were fixed with 4% PFA for 24 h, transferred to 30% sucrose in phosphate-buffered saline (PBS), and preserved at 4 °C. The brains of four mouse fetuses were fixed with 4% PFA for 4 h and preserved using the same method. They were then embedded with O.C.T. compound (Sakura finetek, Torrence, CA, USA) and cut with a cryostat (coronal section of the brain with 50 µm, sagittal section of the brain with 30 µm and longitudinal section of the spinal posterior column lower than C1 level with 30 µm) (CM-1850, Leica Biosystems) and preserved in antifreeze (30% glyceol (Nacalai tesque), 30% ethylene glycol (Wako) and 40% PBS) at −30 °C before use. The cryosections were attached to MAS-coated glass slides (Matsunami, Osaka, Japan). Immunohistochemical analysis of the cryosections was carried out after permeabilization with 0.3% Triton X-100 (Sigma-Aldrich) and blocking in 2% skim milk (BD). The primary antibodies used were anti-GFP (rabbit and rat, 1:1000, MBL International), anti-CTIP2 (rat, 1:500, Abcam), anti-SATB2 (mouse, 1:200, Abcam), anti-PAX6 (rabbit, 1:500, Covance), anti-TBR1 (rabbit, 1:500, Abcam), anti-TBR2 (rabbit, 1:500, Abcam), anti-NRP1 (rabbit, 1:250, ECM Biosciences), anti-FOXG1 (rabbit, 1:1000, Takara Bio Inc.), anti-CUX1 (rabbit, 1:100, Santa Cruz), anti-C-FOS (rabbit, 1:1000, Santa Cruz) and anti-Synaptophysin (guinea pig, 1:1000, Synaptic Systems). For visualization, the secondary antibodies used were Alexa Fluor 488 goat anti-rabbit (1:400, Invitrogen), Alexa Fluor 488 goat anti-rat (1:400, Invitrogen), Alexa Fluor 488 goat anti-guinea pig (1:400, Invitrogen), Alexa Fluor 594 goat anti-rat (1:400, Invitrogen), Alexa Fluor 594 goat anti-mouse (1:400, Invitrogen), Alexa Fluor 594 goat anti-rabbit (1:400, Invitrogen), and Alexa Fluor 647 goat anti-rabbit (1:400, Invitrogen). The tissue samples were mounted on glass slides with fluorescent mounting medium containing 4,6-dimamidino-2-phenylindole (DAPI). We used 20 brain sections per animal for the histological analysis. The analysis was performed using the coronal sections with maximum graft area in all specimens, and we defined GFP/DAPI double-positive cells as engrafted cells.

The immunoreactive cells and fibers were analyzed using a fluorescence microscope (BZ-9000, Keyence) or confocal laser microscope (LSM700, ZEISS; CQ1, YOKOGAWA). The number of positive cells labeled by each primary antibody in the graft and the host brain were counted in the coronal sections. We performed cell counting using Image J (NIH). The original figures in Fig. 1d and Supplementary Fig. 1 were taken by a confocal laser microscope (CQ1, YOKOGAWA). The fluorescence emission was viewed through a dry emersion objective (UPLSAPO20X, Olympus), and Z-stack images were taken in 5 µm intervals over 30 µm. Maximum intensity projection (MIP) images of GFP/DAPI were made using CellPathfinder software (YOKOGAWA) and converted to a tiled figure using Image J.

### Retrograde tracing of transplanted grafts

Fourteen days after the transplantation and TMT, four rats were anesthetized and clamped as described above. Following the incision of the skin overlying a cervical region, laminectomy of C1 was performed, and 0.3 µl of 4% Fast blue (Polysciences, Warrington, PA, USA), and 4% Dimethyl sulfoxide (Sigma-Aldrich, St. Louis, MO, USA) in artificial cerebrospinal fluid (Harvard Apparatus, Holliston, MA, USA) were injected into four sites (0.3 µl/site, vertical 0.5 mm) targeting a posterior column at C1–2 level over 1 min using a sterile 26 gauge needle. Then, 21 days after the transplantation, the rats were transcardially perfusion-fixed and preserved by the same methods.

### Statistical analysis

Statistical analyses were performed using PRISM (GraphPad Software). For the comparison of two groups, the significance of differences was determined by Mann–Whitney test; for the comparison of more than three groups, one-way ANOVA with Tukey’s multiple comparisons test was done. Behavioral data were analyzed with two-way ANOVA with Tukey’s multiple comparisons test. Differences were considered statistically significant when probability values were <0.05. The data are presented as the mean ± standard error of the mean (SEM).

### Reporting summary

Further information on research design is available in the Nature Research Reporting Summary linked to this article.

## Supplementary information

Supplementary Information

Reporting Summary Checklist

## Data Availability

The authors declare that all data supporting the findings of this study are available within the paper and its supplementary information files. Raw data are available from the author upon reasonable request.

## Supplementary information

**Supplementary information** accompanies the paper on the *npj Regenerative Medicine* website (10.1038/s41536-019-0075-6).
