# Supplementary material for: Therapeutic effects of combined cell transplantation and locomotor training in rats with brain injury
Source: NPJ Regen Med. 2019 Jun 5;4:13. doi: 10.1038/s41536-019-0075-6 (PMC6549150; doi:10.1038/s41536-019-0075-6)
Supplement: Supplementary file 1 — Supplementary Information [file 41536_2019_75_MOESM1_ESM.pdf]

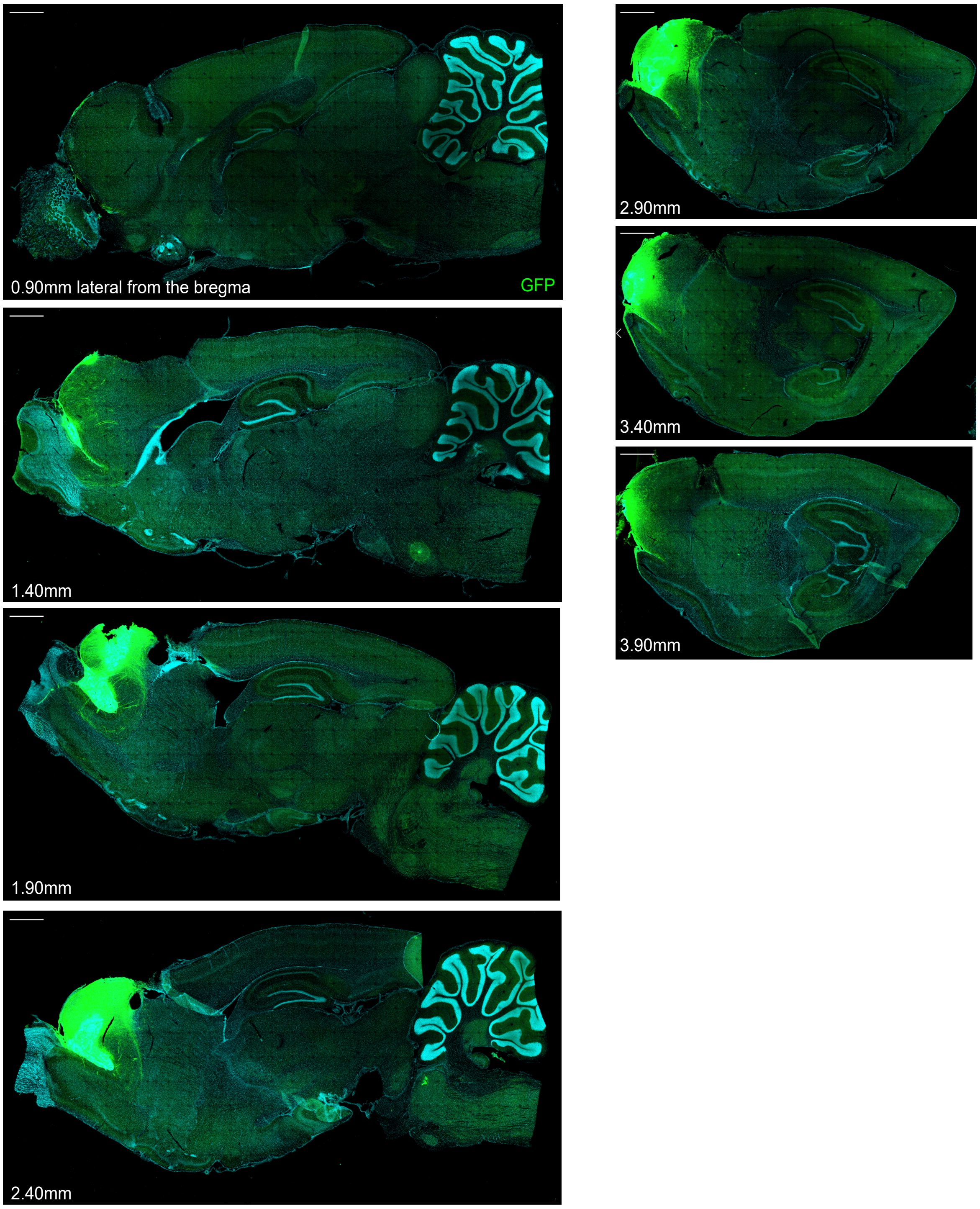

Supplementary Figure 1

E14.5 mouse frontal cortical tissue was engrafted and extended neuronal fibers in a lesioned three-week-old rat brain.

Representative sequential sagittal images of GFP staining at 0.90, 1.40, 1.90, 2.40, 2.90, 3.40 and 3.90 mm lateral from the bregma at two weeks after the cell transplantation. Scale bars, 1000 μm.

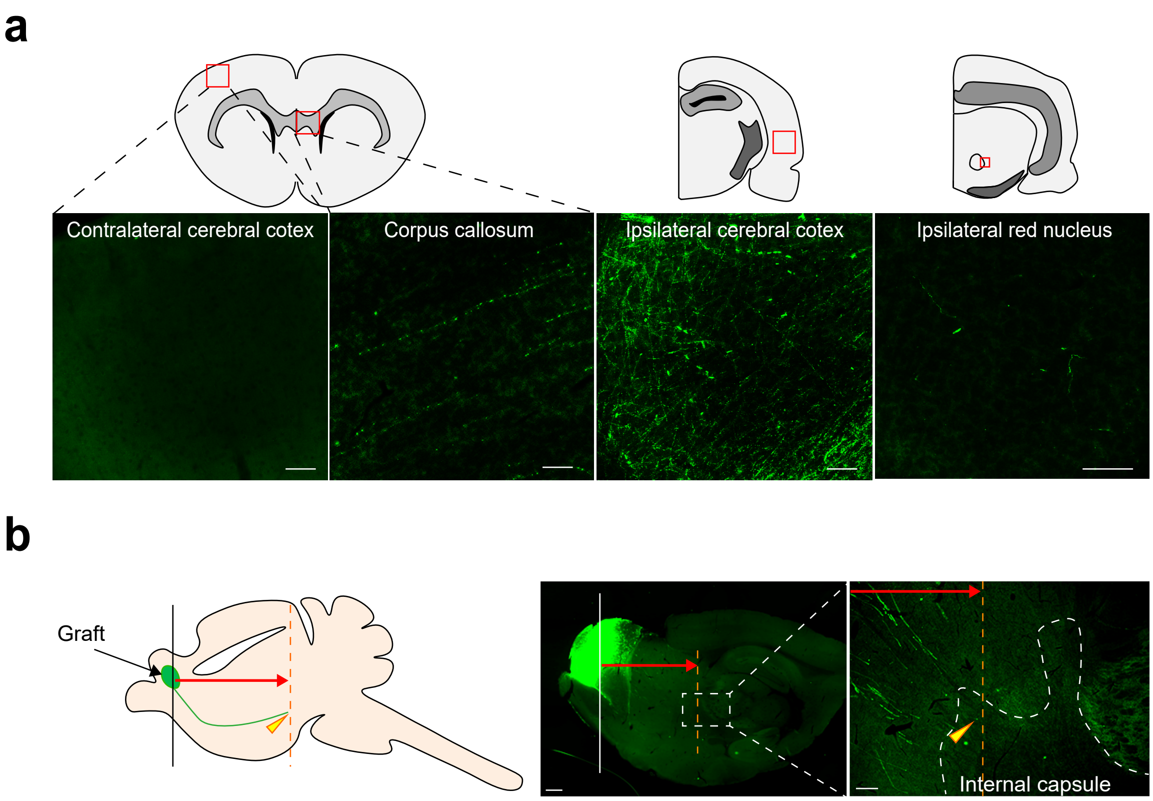

Supplementary Figure 2

TMT promoted neurite extensions from grafted cells.

a) Representative images of extending GFP+ neuronal fibers at the contralateral cortex, the corpus callosum, the ipsilateral cortex and the ipsilateral red nucleus in the TMT (+) group. Scale bars, 50 μm.

b) Schematic of the maximum horizontal distance from the graft to the tip of the longest neurite in the sagittal plane. The red arrow indicates the maximum distance of neuronal growth from the graft. Scale bars, 500 μm (left) and 200 μm (right).

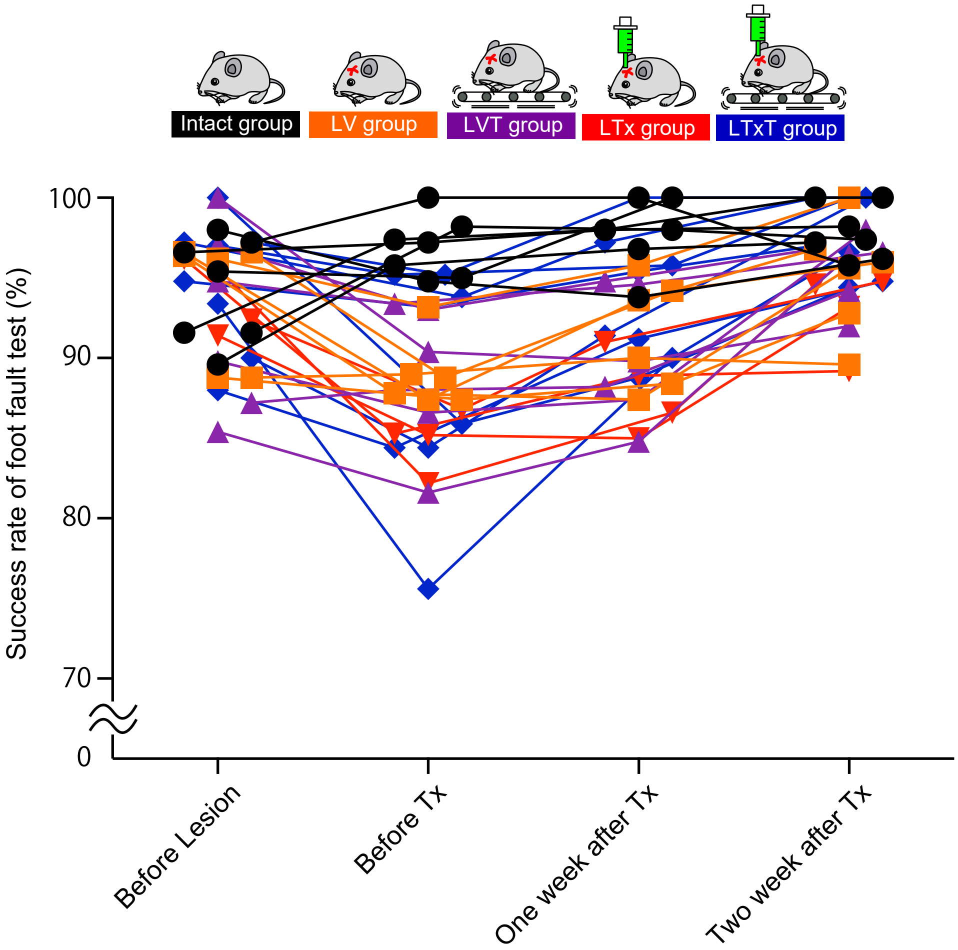

Supplementary Figure 3

Combined therapy might have the potential to promote functional recovery compared to cell transplantation alone.

Serial measurements of the success rate of the foot fault test for the intact group (n=7), lesion and vehicle injection group (LV group; n=6), lesion and vehicle injection with TMT group (LVT group; n=6), lesion and cell transplantation group (LTx group; n=4) and lesion and cell transplantation with TMT group (LTxT group; n=8) are shown. Two-way repeated measures ANOVA.

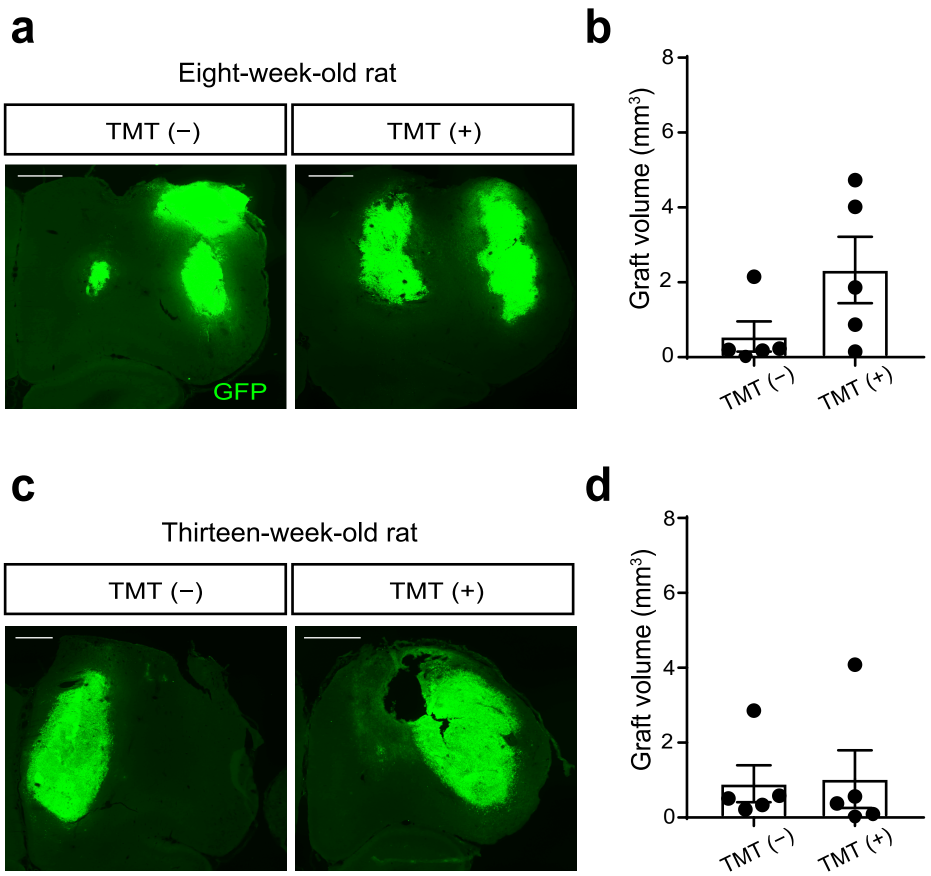

Supplementary Figure 4

Analyses of graft volume in eight-week-old and thirteen-week-old rats.

a) Representative coronal images of GFP staining at two weeks after transplantation in the TMT (−) group and TMT (+) group for eight-week-old rats. Images show a section with maximum graft area. Scale bars, 500 μm.

b) Graft volume. There was no significant difference in graft volumes between groups. Mann-Whitney tests were performed; n=5 in TMT (−) group and n=5 in TMT (+) group. Data are presented as means ± SEM.

c) Representative coronal images of GFP staining at two weeks after cell transplantation in the TMT (−) group and TMT (+) group for thirteen-week-old rats. Images show a section with maximum graft area. Scale bars, 500 μm.

d) Graft volume. There was no significant difference in graft volumes between groups. Mann-Whitney tests were performed; n=5 in TMT (−) group and n=5 in TMT (+) group. Data are presented as means ± SEM.

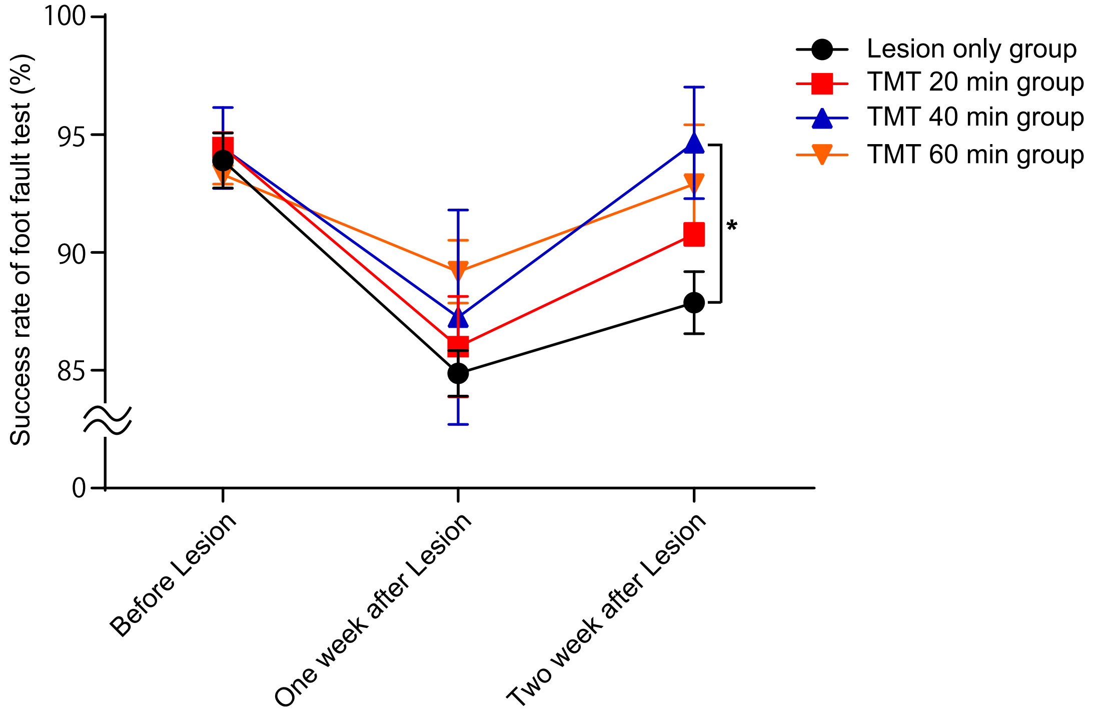

Supplementary Figure 5

Examination to determine the TMT time

Average success rates of the foot fault test for the lesion only group, lesion + 20 min TMT group (TMT 20 min group), lesion + 40 min TMT group (TMT 40 min group) and lesion + 60 min TMT group (TMT 60 min group) are shown (n=4, respectively). Two weeks after the lesion, the success rate was significantly higher in only the TMT 40 min group than the lesion only group. Two-way ANOVA with Tukey' s multiple comparisons test, *$p < 0.05$. Data are presented as means ± SEM.

Supplementary Figure Legends

Supplementary Figure 1

E14.5 mouse frontal cortical tissue was engrafted and extended neuronal fibers in a lesioned three-week-old rat brain.

Representative sequential sagittal images of GFP staining at 0.90, 1.40, 1.90, 2.40, 2.90, 3.40 and 3.90 mm lateral from the bregma at two weeks after the cell transplantation. Scale bars, 1000 µm.

Supplementary Figure 2

TMT promoted neurite extensions from grafted cells.

a) Representative images of extending GFP^{+} neuronal fibers at the contralateral cortex, the corpus callosum, the ipsilateral cortex and the ipsilateral red nucleus in the TMT (+) group. Scale bars, 50 µm.

b) Schematic of the maximum horizontal distance from the graft to the tip of the longest neurite in the sagittal plane. The red arrow indicates the maximum distance of neuronal growth from the graft. Scale bars, 500 µm (left) and 200 µm (right).

Supplementary Figure 3

Combined therapy might have the potential to promote functional recovery compared to cell transplantation alone.

Serial measurements of the success rate of the foot fault test for the intact group (n=7), lesion and vehicle injection group (LV group; n=6), lesion and vehicle injection with TMT group (LVT group; n=6), lesion and

cell transplantation group (LTx group; n=4) and lesion and cell transplantation with TMT group (LTxT group; n=8) are shown. Two-way repeated measures ANOVA.

Supplementary Figure 4

Analyses of graft volume in eight-week-old and thirteen-week-old rats.

a) Representative coronal images of GFP staining at two weeks after transplantation in the TMT (−) group and TMT (+) group for eight-week-old rats. Images show a section with maximum graft area. Scale bars, 500 µm.

b) Graft volume. There was no significant difference in graft volumes between groups. Mann-Whitney tests were performed; n=5 in TMT (−) group and n=5 in TMT (+) group. Data are presented as means ± SEM.

c) Representative coronal images of GFP staining at two weeks after cell transplantation in the TMT (−) group and TMT (+) group for thirteen-week-old rats. Images show a section with maximum graft area. Scale bars, 500 µm.

d) Graft volume. There was no significant difference in graft volumes between groups. Mann-Whitney tests were performed; n=5 in TMT (−) group and n=5 in TMT (+) group. Data are presented as means ± SEM.

Supplementary Figure 5

Examination to determine the TMT time

Average success rates of the foot fault test for the lesion only group, lesion + 20 min TMT group (TMT 20 min group), lesion + 40 min TMT group (TMT 40 min group) and lesion + 60 min TMT group (TMT 60 min group) are shown (n=4, respectively). Two weeks after the lesion, the success rate was significantly

higher in only the TMT 40 min group than the lesion only group. Two-way ANOVA with Tukey’s multiple comparisons test, $*p < 0.05$. Data are presented as means ± SEM.
